# Metabolic Characterization and Consequences of Mitochondrial Pyruvate Carrier Deficiency in *Drosophila melanogaster*

**DOI:** 10.3390/metabo10090363

**Published:** 2020-09-06

**Authors:** Chloé Simard, Andréa Lebel, Eric Pierre Allain, Mohamed Touaibia, Etienne Hebert-Chatelain, Nicolas Pichaud

**Affiliations:** 1 Department of Chemistry and Biochemistry, Université de Moncton, Moncton, NB E1A 3E9, Canada; ecs0175@umoncton.ca (C.S.); eal8075@umoncton.ca (A.L.); mohamed.touaibia@umoncton.ca (M.T.); 2 Atlantic Cancer Research Institute (ACRI), Moncton, NB E1C 8X3, Canada; erica@canceratl.ca; 3 Department of Biology, Université de Moncton, Moncton, NB E1A 3E9, Canada; etienne.hebert.chatelain@umoncton.ca; 4 Canada Research Chair in Mitochondrial Signaling and Physiopathology, Moncton, NB E1A 3E9, Canada

**Keywords:** drosophila, kinetics, metabolomics, mitochondrial pyruvate carrier, mitochondrial respiration

## Abstract

In insect, pyruvate is generally the predominant oxidative substrate for mitochondria. This metabolite is transported inside mitochondria via the mitochondrial pyruvate carrier (MPC), but whether and how this transporter controls mitochondrial oxidative capacities in insects is still relatively unknown. Here, we characterize the importance of pyruvate transport as a metabolic control point for mitochondrial substrate oxidation in two genotypes of an insect model, *Drosophila melanogaster*, differently expressing MPC1, an essential protein for the MPC function. We evaluated the kinetics of pyruvate oxidation, mitochondrial oxygen consumption, metabolic profile, activities of metabolic enzymes, and climbing abilities of wild-type (WT) flies and flies harboring a deficiency in MPC1 (MPC1^def^). We hypothesized that MPC1 deficiency would cause a metabolic reprogramming that would favor the oxidation of alternative substrates. Our results show that the MPC1^def^ flies display significantly reduced climbing capacity, pyruvate-induced oxygen consumption, and enzymatic activities of pyruvate kinase, alanine aminotransferase, and citrate synthase. Moreover, increased proline oxidation capacity was detected in MPC1^def^ flies, which was associated with generally lower levels of several metabolites, and particularly those involved in amino acid catabolism such as ornithine, citrulline, and arginosuccinate. This study therefore reveals the flexibility of mitochondrial substrate oxidation allowing Drosophila to maintain cellular homeostasis.

## 1. Introduction

Insects have the highest metabolic rates in nature and the most rapidly contracting muscles, which makes them relevant model animals for studying metabolic key processes [1,2,3]. Mitochondria resides at the center of these metabolic processes. In flight muscle, mitochondrial respiration and ATP turnover may increase several hundredfold during the transition from rest to flight. Thus, to sustain these high levels of activity, insect flight muscle metabolism must be extremely flexible. Flexibility is also required according to the type and abundance of fuels that are oxidized [3]. In most species of Diptera such as *Drosophila melanogaster*, carbohydrate constitutes the predominant mitochondrial substrate sustaining the high level of oxygen consumption and ATP production required for flight [2,4].

Under physiological conditions, carbohydrates such as glucose derived from either glycogen or trehalose (the most important circulating carbohydrate in insects) are processed by the glycolysis in the cytosol, which produces important metabolites such as pyruvate and ATP. Pyruvate can then cross the inner mitochondrial membrane via the mitochondrial pyruvate carrier (MPC), a widely conserved protein constituted by two essential proteins, MPC1 and MPC2 [5]. Pyruvate is then further metabolized in the tricarboxylic acid (TCA) cycle, producing the reducing equivalents (NADH and FADH_2_) necessary for the transfer of electrons into the electron transport system (ETS). The ETS protein complexes (complexes I to IV) embedded in the mitochondrial inner membrane then use these reducing equivalents as electron donors which are transferred from one complex to the other, until the final acceptor of the system is reached, i.e., dioxygen. This electron transport generates a proton gradient (at the level of complexes I, III, and IV) that is then used to fuel the ATP synthase (complex V) to produce ATP by the oxidative phosphorylation (OXPHOS) process.

Alternative fuels can also be used in insects to sustain mitochondrial respiration [6]. It has been shown that proline and glycerol-3-phosphate (G3P) can serve as such oxidative substrates participating in electron transport, increasing mitochondrial oxygen consumption in several insect species, including *Drosophila* [4,7,8,9]. While most animals avoid relying on proteins, some insects use the amino acid proline as their main fuel [8]. This is the case for the tsetse fly [10], the Colorado potato beetle [11], as well as bumblebees and wasps [8], among others. Proline can either be oxidized in combination with carbohydrates, playing an anaplerotic role by increasing TCA intermediates to oxidize pyruvate at high rates, or directly oxidized by the mitochondrial proline dehydrogenase (ProDH) to provide electrons to the ETS [8,9,12,13,14]. Additionally, the mitochondrial glycerol-3-phosphate dehydrogenase (mtG3PDH) allows electrons to enter the ETS through the reduction of glycerol-3-phosphate derived from either dihydroxyacetone phosphate (a metabolite from glycolysis) or from the glycerol obtained by triglyceride or diglyceride degradation [5,6,15]. Thus, G3P production and mitochondrial oxidation are linked to both carbohydrate and lipid metabolism. It is therefore crucial to understand the regulatory mechanisms at the three mitochondrial loci of metabolic control in insects; namely, the oxidation of pyruvate, proline, and G3P.

Amongst all these different pathways and metabolites necessary for the proper functioning of mitochondrial metabolism, pyruvate is a key molecule. First, it is central to glucose metabolism and homeostasis, as it is the end-product of glycolysis. As such, it is also a critical intermediate of aerobic and anaerobic metabolism, gluconeogenesis, TCA cycle, and amino acid metabolism. The cytosolic pyruvate pool comes mostly from the breakdown of glucose through glycolysis. Pyruvate can also be produced by the oxidation of lactate and several amino acids. One of the most important fates of pyruvate is to be transported into the mitochondria for non-reversible transformation by pyruvate dehydrogenase (PDH) into acetyl-CoA, which sparks the TCA cycle. It has been shown that the inhibition of mitochondrial transport of pyruvate in *Drosophila* mutants with deleted MPC1 alleles causes increased levels of glycolytic intermediates, glucose intolerance, and high levels of circulating glucose and trehalose [16,17]. However, considering the different substrates that can act as electron providers to the mitochondrial ETS in insects, a proper evaluation of mitochondrial metabolism in *Drosophila* harboring a deficiency in pyruvate transport is required to draw conclusions about the specific consequences of such alteration.

In this study, we investigated the importance of pyruvate import into mitochondria in two genotypes of an insect model, *Drosophila melanogaster*, differently expressing MPC1. Specifically, we determined the kinetics of pyruvate oxidation, mitochondrial oxygen consumption using different oxidative substrates, metabolic profile using ^1^H nuclear magnetic resonance (NMR) spectroscopy, enzymatic activities of pyruvate kinase (PK), lactate dehydrogenase (LDH), alanine aminotransferase (ALAT), pyruvate dehydrogenase (PDH), citrate synthase (CS) and hydroxyacyl Co-enzyme A dehydrogenase (HOAD), as well as the climbing abilities of wild-type (WT) flies and flies harboring a mild deficiency in MPC1 (MPC1^def^). Considering the importance of carbohydrate metabolism in insects, we hypothesized that a MPC1 deficiency would cause an accumulation of glycolysis intermediates and a metabolic reprogramming that would favor the oxidation of alternative substrates to maintain mitochondrial respiration and restore cell homeostasis. In other words, if the oxidation of pyruvate is impaired due to defects in mitochondrial transport, the mitochondrial locus determining substrate oxidation switches to either proline or G3P oxidation, leading to major consequences at the level of overall metabolism.

## 2. Results

### 2.1. MPC1 Expression and Mitochondrial Pyruvate Carrier Inhibition Kinetics

**Figure 1:**
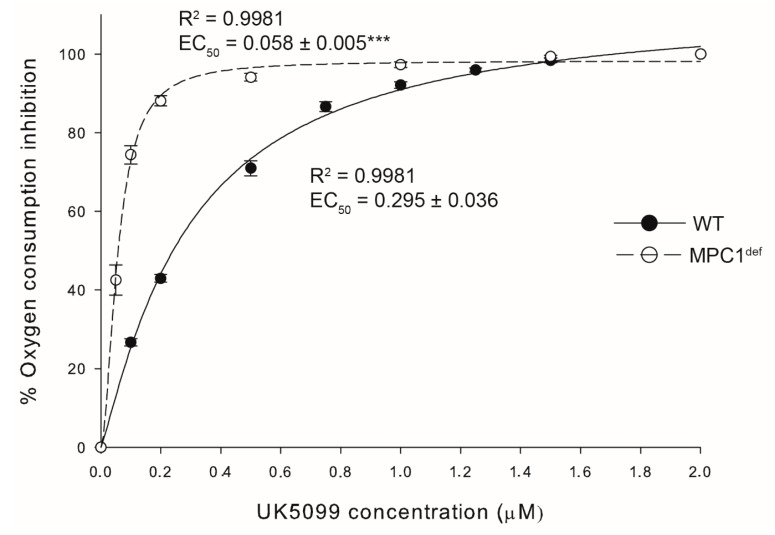
Kinetics of mitochondrial pyruvate oxidation measured in permeabilized thorax of WT and MPC1^def^ *Drosophila melanogaster*. Mitochondrial oxygen consumption was measured in presence of pyruvate (10 mM) and ADP (5 mM) and following successive injections of the MPC inhibitor UK-5099 (0 to 2 µM) until complete inhibition was achieved (N = 6). The percentage of pyruvate-induced oxygen consumption inhibition was plotted against the UK-5099 concentration, and a four-parameter logistic curve was fitted to estimate the EC_50_ (half maximal effective concentration) for both genotypes. Results are means ± SEM. R^2^ represents the coefficient of determination for the fitting curve. *** denotes significant differences between the EC_50_, with *p* < 0.001.

The MPC1^def^ flies showed approximately a 2-fold decrease of *MPC1* expression relative to WT with either *α-tubulin* (1.07 ± 0.20 and 0.59 ± 0.33 for WT and MPC1^def^, respectively) or *rpl32* (1.01 ± 0.11 and 0.55 ± 0.33 for WT and MPC1^def^, respectively) as reference genes. To ascertain that this mild deficiency caused mitochondrial dysfunctions, progressive inhibition of pyruvate-induced mitochondrial oxygen consumption during OXPHOS respiratory state (when ADP was added with pyruvate to trigger the phosphorylation of ADP to ATP) was performed in both genotypes using increasing concentrations of UK-5099 (Millipore-Sigma), a well-known inhibitor of pyruvate transport [18]. For both genotypes, the plotted four parameter logistic curve presented very good fits (R^2^ = 0.998 for both genotypes; Figure 1). The calculated EC_50_ values were 295 nM and 58 nM for WT and MPC1^def^ flies, respectively, and were significantly different between genotypes (*p* < 0.001; Figure 1). Altogether, these results show that MPC1^def^ flies have a genomic deficiency that translates into a functional deficiency at the mitochondrial level.

### 2.2. Mitochondrial Oxygen Consumption and Mitochondrial Ratios

Mitochondrial oxygen consumption was measured at several steps of the ETS, according to a method described by Simard et al. (2018). Briefly, CI-LEAK represents the oxygen consumption used to compensate for the proton leak at the level of complex I when ATP synthase is not active (due to the absence of ADP) but the reducing substrate pyruvate is present. CI_pyr_-OXPHOS, CI_pyr + mal_-OXPHOS, CI + ProDH-OXPHOS, CI + ProDH + CII-OXPHOS, and CI + ProDH + CII + mtG3PDH-OXPHOS represent the oxygen consumption during the OXPHOS state (with the presence of ADP) with different substrates (pyruvate, malate, proline, succinate, and G3P, respectively) to stimulate the transport of electrons from the different complexes of the ETS. This respiratory state is characterized by the oxidation of reduced fuel substrates allowing electron transfer to oxygen, which is chemiosmotically coupled to the phosphorylation of ADP to ATP. In this respiratory state, the substrates proline, succinate, and G3P provide electrons to proline dehydrogenase, complex II, and the mitochondrial glycerol-3-phosphate dehydrogenase, respectively, thus increasing electron transfer to oxygen, ADP phosphorylation, and oxygen consumption. Finally, CI + ProDH + CII + mtG3PDH-ETS represents the noncoupled respiration after the addition of the uncoupler FCCP i.e., the non-phosphorylating respiration stimulated to maximal oxygen consumption. During this state, the electron transfer is not chemiosmotically coupled to the phosphorylation of ADP to ATP, and the electron transport to oxygen is maximized because it is not limited by the ATP synthase.

**Figure 2:**
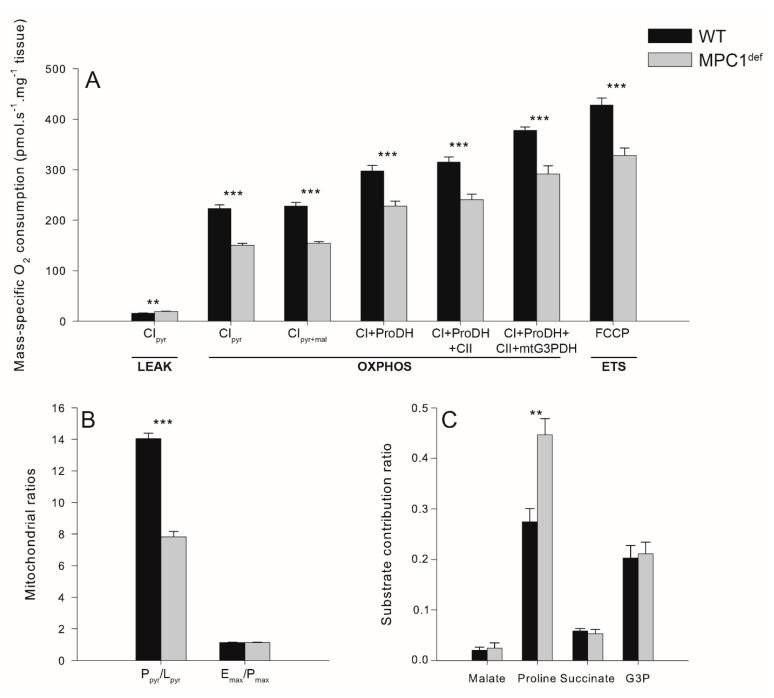
Mass-specific oxygen consumption rates and mitochondrial ratios measured in permeabilized thorax of WT and MPC1^def^ *Drosophila melanogaster*. (**A**) Mitochondrial oxygen consumption rates were measured in presence of pyruvate (CI_pyr_-LEAK), pyruvate + ADP (CI_pyr_-OXPHOS), pyruvate + ADP + malate (CI_pyr + mal_-OXPHOS), pyruvate + ADP + malate + proline (CI + ProDH-OXPHOS), pyruvate + ADP + malate + proline + succinate (CI + ProDH + CII-OXPHOS), pyruvate + ADP + malate + proline + succinate + G3P (CI + ProDH + CII + mtG3PDH-OXPHOS), pyruvate + ADP + malate + proline + succinate + G3P + FCCP (ETS); (**B**) Calculated P_pyr_/L_pyr_ ratio (CI-OXPHOS/CI-LEAK) and E_max_/P_max_ ratio (CI + ProDH + CII + mtG3PDH-ETS/CI + ProDH + CII + mtG3PDH-OXPHOS); (**C**) Substrate contribution calculated as: malate contribution = ((CI_pyr + mal_-OXPHOS − CI_pyr_-OXPHOS)/CI_pyr_-OXPHOS), proline contribution = ((CI + ProDH-OXPHOS − CI_pyr + mal_-OXPHOS)/CI_pyr + mal_-OXPHOS), succinate contribution = ((CI + ProDH + CII-OXPHOS − CI + ProDHOXPHOS)/CI + ProDHOXPHOS), G3P contribution = ((CI + ProDH + CII + mtG3P-OXPHOS − CI + ProDH + CII-OXPHOS)/CI + ProDH + CII-OXPHOS). Results are means ± SEM for each genotype (N = 6), with stars denoting statistical differences: ** *p* < 0.01, *** *p* < 0.001.

WT flies had significantly lower CI_pyr_-LEAK than MPC1^def^ flies (*p* = 0.001; Figure 2A). However, for all the other respiration rates measured (CI_pyr_-OXPHOS, CI_pyr + mal_-OXPHOS, CI + ProDH-OXPHOS, CI + ProDH + CII-OXPHOS, CI + ProDH + CII + mtG3PDH-OXPHOS and CI + ProDH + CII + mtG3PDH-ETS), the WT flies had significantly higher mitochondrial oxygen consumption than the MPC1^def^ flies (all *p*-values < 0.001; Figure 2A). Increased CI_pyr_-LEAK and decreased CI_pyr_-OXPHOS in MPC1^def^ flies resulted in a significantly lower P_pyr_/L_pyr_ ratio (indicative of mitochondrial quality and of mitochondrial coupling [19]) than WT flies (*p* < 0.001; Figure 2B), suggesting that pyruvate-induced oxygen consumption is impaired in MPC1^def^ flies. The E_max_/P_max_ ratio was not different between genotypes and was not significantly different from 1.0 (1.13 ± 0.019 and 1.13 ± 0.017 for WT and MPC1^def^, respectively), denoting that the phosphorylation system did not limit oxygen consumption in either genotype with the combination of substrates used (Figure 2B).

The different contribution of each substrate injected to stimulate the electron flux into the ETS, and hence mitochondrial oxygen consumption, was then calculated from the different respiration rates measured. After injection of malate, very small increases of mitochondrial oxygen consumption were measured without significant differences between genotypes (around 2% increase; Figure 2C). However, when proline was injected, mitochondrial oxygen consumption increased by around 25% in WT and 45% in MPC1^def^, with significant differences between genotypes (*p* = 0.002; Figure 2C). Succinate and G3P also triggered increased mitochondrial oxygen consumption (around 5.5% and 20%, respectively) without significant differences being detected between genotypes (Figure 2C).

### 2.3. Enzymatic Activities

**Figure 3:**
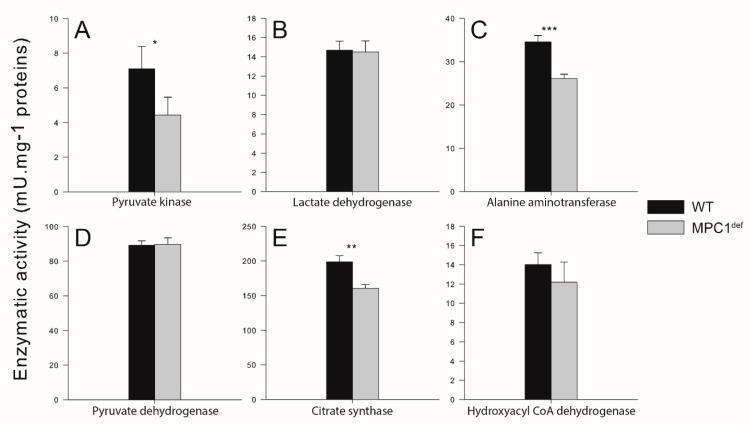
Enzymatic activities measured in thorax of *Drosophila melanogaster* WT and MPC1^def^. (**A**) Pyruvate kinase activity; (**B**) Lactate dehydrogenase activity; (**C**) Alanine aminotransferase activity; (**D**) Pyruvate dehydrogenase activity; (**E**) Citrate synthase activity; (**F**) Hydroxyacyl-Coenzyme A dehydrogenase activity. Results are means ± SEM for each genotype (N = 6), with stars denoting statistical differences: * *p* < 0.05, ** *p* < 0.01, *** *p* < 0.001.

For the glycolytic enzymes producing and consuming pyruvate (PK and LDH, respectively; Figure 3A,B), only PK was different between genotypes, with WT flies having higher levels of activity than MPC1^def^ flies (*p* = 0.031; Figure 3A). The ALAT, which reversibly converts pyruvate to alanine, was also significantly lower in MPC1^def^ flies (*p* < 0.001; Figure 3C). Although PDH, which converts pyruvate to acetyl-CoA for further oxidation in the TCA cycle, was not different between genotypes (Figure 3D), CS, which mediates the conversion of acetyl-CoA and oxaloacetate to citrate, was significantly lower in MPC1^def^ flies (*p* = 0.003; Figure 3E). Finally, HOAD, involved in fatty acid catabolism, was not different between genotypes (Figure 3F). 

### 2.4. Relative Metabolites Abundance

**Figure 4:**
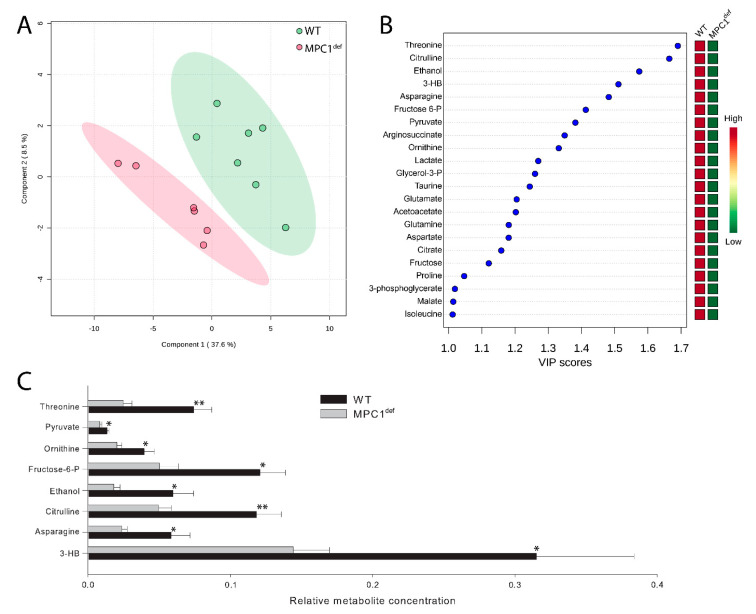
Metabolite profile analysis in *Drosophila melanogaster* WT and MPC1^def^. (**A**) PLS-DA score plots of metabolites in WT and MPC1^def^ flies. Ellipses represent 95% confidence intervals for each individual group on PLS-DA plots with the variance proportion represented by components 1 and 2. (**B**) Variable importance of projection (VIP) scores of PLS-DA for component 1, which identify the key metabolites driving the metabolomic signature for WT and MPC1^def^ flies. (**C**) Relative metabolite abundance identified as drivers of the metabolomic profile of WT and MPC1^def^ flies (VIP > 1.3). Data are presented as means ± SEM (N = 6–7) with stars denoting statistical differences: * *p* < 0.05, ** *p* < 0.01.

Relative metabolite abundance was analyzed using the multivariate PLS-DA [20] to evaluate potential metabolic shifts due to impairment of mitochondrial pyruvate import (Figure 4A). The two main PLS components explained 37.6% and 8.5% of the total variance, respectively (Figure 4A).

WT flies separated from MPC1^def^ flies on the first PLS component, showing that the genotypes have a different metabolic signature (Figure 4A). Variable importance of projection (VIP) scores identified 22 metabolites significantly driving the specific metabolomic signature between genotypes (i.e., metabolites with VIP > 1; Figure 4B and Table S2). All these key metabolites were generally decreased in MPC1^def^ flies compared to WT flies (Figure 4B). However, only eight of these metabolites were significantly different between genotypes (threonine, *p* = 0.008; pyruvate, *p* = 0.028; ornithine, *p* = 0.039; fructose 6-phosphate, *p* = 0.011; ethanol, *p* = 0.026; citrulline, *p* = 0.007; asparagine, *p* = 0.041; and 3-hydroxybutyrate, *p* = 0.049; Figure 4C).

### 2.5. Climbing Assay

**Figure 5:**
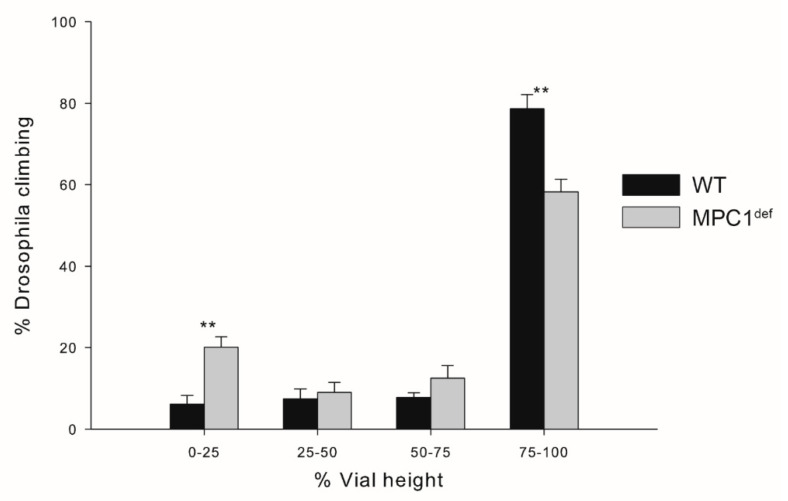
Climbing abilities of *Drosophila melanogaster* WT and MPC1^def^. The number of flies climbing in the four different quarters of the vial (0–25, 25–50, 50–75, and 75–100% of the total height of the vial) was counted after 30 s for each genotype. Stars depict significant differences between the genotypes with ** *p* < 0.01 (N ≥ 75 for each genotype).

WT flies had a greater capacity to climb the vial compared to MPC1^def^ flies. Specifically, significantly fewer WT flies were recorded in the lower quadrant (6.12 ± 2.14% and 20.16 ± 2.52% for WT and MPC1^def^, respectively, *p* = 0.001; Figure 5), and significantly more were observed in the upper quadrant (78.64 ± 3.47% and 58.20 ± 3.17% for WT and MPC1^def^, respectively, *p* = 0.001; Figure 5).

## 3. Discussion

**Figure 6:**
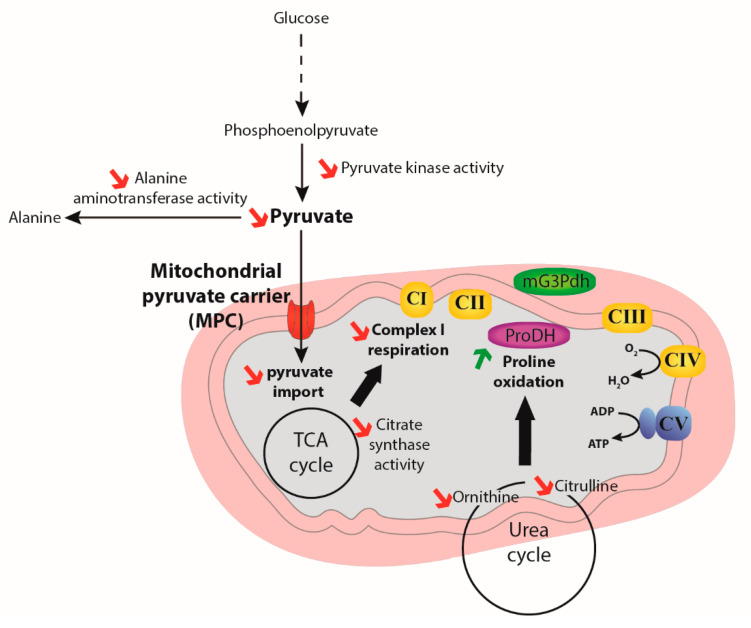
Schematic representation of the effects of decreased mitochondrial pyruvate transport in MPC1^def^ Drosophila.

In this study, we sought to characterize the importance of pyruvate import into mitochondria as a metabolic control point for mitochondrial substrate oxidation in two genotypes of an insect model, *Drosophila melanogaster*, differently expressing MPC1. Our results show that the MPC1^def^ flies displayed a general reduction of metabolic functions. Specifically, significantly reduced climbing capacity, pyruvate-induced oxygen consumption, and enzymatic activities of PK, ALAT, and CS were detected in MPC1^def^ flies compared to WT flies (Figure 6). Moreover, increased proline oxidation capacity was detected in MPC1^def^ flies, which was associated with generally lower levels of several metabolites, and particularly those involved in amino acid catabolism (Figure 6). This reveals the crucial role of MPC in insects as a node controlling the flexibility of substrate oxidation by mitochondria and the importance of such flexibility for the physiology of organisms.

Considering that pyruvate is the main substrate derived from carbohydrate metabolism allowing mitochondrial respiration, impairment of its transport in insects should inevitably result in reduced mitochondrial oxygen consumption in the thorax muscles, as well as deleterious effects at the organismal level. Our results show that the MPC1^def^ flies had a two-fold decrease in *MPC1* expression that translated into a drastic reduction of the calculated EC_50_ for a specific inhibitor of MPC (58 nM and 295 nM for MPC1^def^ and WT flies, respectively). MPC1^def^ flies also displayed significantly lower mitochondrial oxygen consumption than WT flies when pyruvate was used as the oxidative substrate. These results are in accordance with several studies showing that MPC1 and/or MPC2 deficiencies impair mitochondrial pyruvate oxidation in mammalian cells [16,21,22,23]. The MPC1^def^ flies also displayed a higher CI_pyr_-LEAK than WT flies, resulting in significantly decreased P_pyr_/L_pyr_ ratios, and hence, further confirming decreased mitochondrial capacity. Moreover, the activity of CS, a good proxy for aerobic metabolism, was significantly decreased in MPC1^def^ flies. These results suggest that impairment of pyruvate import via MPC affects the aerobic capacity of flies and the ability of mitochondria to use pyruvate as metabolic fuel. CS activity can also be used as an indicator of mitochondrial content [24]. Thus, an alternative explanation is that the overall reduction of mitochondrial respiration observed in MPC1^def^ flies might be due to a decrease of mitochondrial content. However, the CS activity may be differentially regulated in the different genotypes, which may indicate changes at the level of enzyme molecular abundance/activity, rather than changes in mitochondrial content. Moreover, the activity of two other mitochondrial enzymes, PDH and HOAD, were not different between genotypes. Thus, it is unlikely that MPC1^def^ flies have different mitochondrial content than WT flies.

As pyruvate is located at the crossroads between an oxidative route involving mitochondria (aerobic metabolism) and a fermentative route (anaerobic metabolism), it is possible that the decreased mitochondrial oxygen consumption induced by pyruvate in MPC1^def^ flies results in increased anaerobic capacity. It has been shown that homozygous mutant mice expressing a truncated form of the MPC2 protein have elevated blood lactate, suggesting that a higher proportion of pyruvate is converted to lactate [25]. Moreover, impaired import of pyruvate via loss of the MPC has also been shown to enforce the Warburg effect in cancer cells [23]. Since climbing muscles favor anaerobic metabolism in Drosophila [26,27], climbing ability was measured to determine whether anaerobic muscle function would be influenced by mitochondrial pyruvate transport. Our results showed that MPC1^def^ flies had a decreased climbing ability compared to WT flies, thus suggesting that their anaerobic capacity was not enhanced following impairment of pyruvate import. LDH activity was not different between genotypes, further confirming that the MPC1^def^ flies did not rely on anaerobic metabolism more than WT flies. Other end-products derived from pyruvate, including lactate, alanine, and acetate, can accumulate during anaerobic metabolism in Drosophila [28,29]. Here, acetate was similar between genotypes (0.070 ± 0.013 and 0.071 ± 0.005 mM in WT and MPC1^def^, respectively), and lactate and alanine were slightly but not significantly lower in MPC1^def^ flies. Furthermore, the activity of ALAT was significantly decreased in MPC1^def^ flies. Altogether, these results further confirm that MPC1^def^ flies display impairment of aerobic and, to a lesser extent, anaerobic capacities.

Interestingly, PK activity and several metabolites of glycolysis were significantly decreased in MPC1^def^ flies. Specifically, reduced levels of pyruvate and fructose-6-phosphate were detected in MPC1^def^ flies. Moreover, levels of trehalose were identical in both genotypes and, although glucose and glycogen were slightly lower in MPC1^def^ flies, all these metabolites were ascribed low VIP scores. These results contradict our hypothesis, as well as other studies showing that MPC1 deletion causes accumulation of glycolysis intermediates [16,17]. However, the flies used in our study presented only a mild deficiency of MPC1, which might explain these discrepancies. Moreover, it is possible that the P-element insertion in MPC1^def^ flies caused other unknown mutations, which could have biased our results. However, considering the effects of the MPC1 deficiency on mitochondrial pyruvate oxidation, we rationalize that the main effects observed here are due to this specific deficiency. The decreased PK activity might also partially explain the reduced pyruvate-induced mitochondrial respiration observed here and could suggest that the whole glycolytic pathway is reduced in MPC1^def^ flies. 

In *Drosophila*, proline increases mitochondrial oxygen consumption at a much lower rate than in bumblebees and wasps (15–25% and 50% increases for Drosophila and bumblebees/wasps, respectively) [5,8]. However, the contribution of proline to mitochondrial respiration was close to 45% in MPC1^def^ flies, suggesting that the MPC1 deficiency could induce a metabolic reprogramming favoring the mitochondrial oxidation of amino acids, instead of carbohydrates generated by the glycolysis. Proline oxidation has been suggested to help replenish TCA cycle intermediates and to increase pyruvate oxidation rates. Levels of TCA cycle intermediates were, however, similar or lower in MPC1^def^ flies compared to WT flies (Table S2), which suggests that proline oxidation directly acts as an electron donor to the ETS. Proline metabolism is tightly linked to the urea cycle and can be produced via glutamate and ornithine in animals, including insects [30,31,32,33]. Interestingly, three major metabolites of the urea cycle (ornithine, citrulline and arginosuccinate with VIP scores of 1.33, 1.66 and 1.35, respectively; Table S2) significantly drive the variation between the genotypes, and are present at reduced levels in MPC1^def^ flies (as well as proline). Similarly, the amino acids threonine, asparagine, glutamate, and aspartate that can be oxidized by the TCA cycle are also important in explaining the metabolic variation between genotypes (VIP scores of 1.69, 1.48, 1.20 and 1.18, respectively), with significantly reduced levels of threonine and asparagine in MPC1^def^ flies. This suggests that amino acid catabolism and proline biosynthesis might be used to compensate the pyruvate-induced mitochondrial respiration deficiency observed in MPC1^def^ flies.

G3P can also be used as an oxidative substrate in *Drosophila* to increase mitochondrial respiration [4,7,34,35]. Recently, it has been shown that decreased mitochondrial respiration at the level of complex I induced by mitonuclear incompatibilities was associated with an increase in the mitochondrial G3P oxidation, compensating for complex I deficiency [36]. It is therefore possible that the decreased pyruvate-induced mitochondrial respiration observed in MPC1^def^ flies leads to an increased contribution of the mtG3PDH to compensate for this deficiency. Although G3P levels were slightly but not significantly reduced in MPC1^def^ flies, the G3P contribution was similar between the two genotypes tested. G3P levels may be associated with triglyceride or diglyceride degradation, forming fatty acids which are further oxidized in mitochondria. However, an essential enzyme of fatty acid oxidation HOAD showed similar activity between WT and MPC1^def^ flies. The reduced levels of G3P observed here might thus reflect lower rates of glycolysis in MPC1^def^ flies, as this substrate can also be derived from the glycolytic intermediate dihydroxyacetone phosphate. Levels of 3-hydroxybutyrate (3-HB) were also significantly lower in MPC1^def^ flies, which suggests that fatty acid metabolism might also be affected in these flies. However, we do not have enough results on fatty acid metabolism to draw conclusions about this.

In summary, our results suggest that a mild genomic deficiency of MPC1 in *Drosophila* leads to major shifts in the overall metabolism. Specifically, MPC1^def^ flies display evidence of reduced glycolysis and mitochondrial respiration induced by carbohydrate derived-substrates. However, the mitochondrial deficiency to transport and oxidize pyruvate might be compensated for by increased reliance on proline for mitochondrial respiration. This is likely due to a metabolic reprogramming favoring amino acid catabolism instead of carbohydrate metabolism to sustain mitochondrial respiration. Thus, MPC is an important metabolic control point for mitochondrial substrate oxidation, as a mild deficiency of pyruvate import can reshape the mitochondrial metabolism to oxidize alternative substrates. Considering that *Drosophila* mostly rely on carbohydrates to fuel metabolism, this might have drastic consequences at the phenotypic level and on longevity. For example, it has been shown that transheterozygotes MPC1 Drosophila have shorter lifespans than controls when exposed to either low or high sugar diets [17]. Other studies have shown that decreased expressions of other metabolite transporters result in lifespan extension in Drosophila. For example, decreased expression of the *Indy* gene, which is associated with mitochondrial citrate transport, results in increased lifespans in flies, as well as in worms and mice [37,38,39]. Future studies evaluating overall fitness and longevity are therefore required to draw conclusions about the effects of reduced pyruvate transport in our model. However, as mitochondrial substrate oxidation, as well as longevity, depend on the type and abundance of dietary macronutrients, it is possible that this enhanced amino acid catabolism might not be observed when higher carbohydrate levels are present in the diet. Moreover, the MPC1^def^ flies used in this study only presented a mild pyruvate transport deficiency, which still allows pyruvate to cross the inner mitochondrial membrane, and were generated using P-element insertion that could have resulted in other unknown mutations. Future research investigating the effects of the dietary protein:carbohydrate ratio [40,41,42] in combination with severe MPC deficiencies or deletions generated using either precise excision of the P-element or CRISPR/Cas9 system could provide a better understanding of this metabolic reprogramming, and of the importance of pyruvate import into mitochondria as a metabolic control point for mitochondrial substrate oxidation in *Drosophila*.

## 4. Materials and Methods

### 4.1. Drosophila

Drosophila melanogaster genotypes w^1118^ and w[1118]; P{w[+mC] = XP}Mpc1[d00809], hereafter referred as WT and MPC1^def^, respectively, were obtained from the Bloomington Drosophila Stock Center (Bloomington, IN, USA). Flies were fed with standard cornmeal medium containing a mixture of 5 g of agar, 6 g of table sugar, 27 g of dried yeast, and 53 g of cornmeal flour dissolved in 1 L tap water. Propionic acid (4 mL) and methyl-4-hydroxybenzoate 10% (*w*/*v*) in ethanol (16 mL) were added to avoid mite and mold contamination. Drosophila were reared at constant density (15 flies for approximately 5 mL of medium), temperature (24.0 ± 0.1 °C), humidity and diurnal cycle (12:12). For each experiment, male flies were collected at 15 days of age, and were either directly processed or flash frozen and kept at −80 °C for subsequent experiments.

### 4.2. MPC1 Expression

The genotype w[1118]; P{w[+mC] = XP}Mpc1[d00809] (MPC1^def^) was generated using P-element insertion [39]. These insertions frequently interfere directly with gene function and can also be remobilized to generate mutations via imprecise excision [40,41]. To evaluate whether the insertion caused changes in *MPC1* expression, a pool of male flies at different ages were sampled and *MPC1* transcript levels were evaluated in the thorax (N = 3–4) after total RNA extraction using TRIzol reagent (Millipore-Sigma, Oakville, ON, Canada) according to the manufacturer’s protocol. Briefly, the 260/280 nm and 260/230 nm absorbance ratios were used to verify the quality of the RNA in each sample. Total RNA (1 µg) was reverse transcribed using the SensiFAST™ cDNA synthesis kit (Bioline, London, UK). Real-time quantitative PCR was performed on a CFX Connect^TM^ (Biorad, Mississauga, ON, Canada) by incubating the cDNA with forward and reverse primers for *MPC1*, *α-tubulin* and *rpl32* (Table S1) and using the SensiFAST™ SYBR^®^ No-ROX kit (Bioline, London, UK) using the following protocol: denaturation for 2 min at 95 °C, followed by 34 cycles of 5 s at 95 °C and 30 s at 55 °C. Results were analyzed using the ΔΔCq method, with the reference gene being either *α-tubulin* or *rpl32*.

### 4.3. Mitochondrial Pyruvate Carrier Inhibition

To evaluate the chemical inhibition of pyruvate import, UK-5099 (Millipore-Sigma, Oakville, ON, Canada) was used to progressively inhibit the pyruvate-induced mitochondrial oxygen consumption during OXPHOS [18]. Permeabilization of thorax and measurement of mitochondrial oxygen consumption at 24 °C (N = 6 for each genotype) were performed as previously described [42]. Briefly, mitochondrial oxygen consumption was measured using an Oxygraph-O2K (Oroboros Instruments, Innsbruck, Austria) filled with an air-saturated respiration medium in the presence of pyruvate (10 mM) and ADP (5 mM) and following successive injections of UK-5099 (0 to 2 µM) until complete inhibition was achieved. The percentage of pyruvate-induced oxygen consumption inhibition was plotted against the UK-5099 concentration, and a four-parameter logistic curve was fitted to estimate the EC_50_ (half maximal effective concentration) for both genotypes.

### 4.4. Mitochondrial Oxygen Consumption

Mitochondrial oxygen consumption at 24 °C (N = 6 for each genotype) in permeabilized thorax was estimated using a SUIT protocol [42,43]. First, the LEAK respiration with pyruvate (10 mM) at the level of complex I was measured (CI_pyr_-LEAK). Injection of ADP (5 mM) enabled the measurement of mitochondrial oxygen consumption when the transport of electrons from complex I was coupled to the phosphorylation of ADP to ATP (CI_pyr_-OXPHOS). These respiration rates were used to calculate the P_pyr_/L_pyr_ ratio using pyruvate as the oxidative substrate to supply electrons at the level of complex I (CI_pyr_-OXPHOS/CI_pyr_-LEAK), indicative of mitochondrial quality and of mitochondrial coupling [19]. Malate (2mM) was added to maintain the carbon flux into the TCA cycle (CI_pyr + mal_-OXPHOS), and the functional integrity of the outer mitochondrial membrane was then verified by the addition of cytochrome *c* (15 μM, CIc-OXPHOS) [42,44]. All preparations tested demonstrated structural integrity of the outer mitochondrial membrane (results not shown). Subsequent injections of several substrates were then performed: proline (5 mM), an important substrate for mitochondrial respiration in insects [7] which provides electrons to the ETS via the proline dehydrogenase (CI + ProDH-OXPHOS); succinate (20 mM), which brings electrons to the ETS through complex II (CI + ProDH + CII-OXPHOS); and G3P (15 mM), that allows the transport of electrons to the ETS via the mtG3PDH (CI + ProDH + CII + mtG3PDH-OXPHOS). The respiration rates measured with these different substrates were used to evaluate the contribution of each substrate to mitochondrial oxygen consumption during OXPHOS: (i) malate contribution = (CI_pyr + mal_-OXPHOS − CI_pyr_-OXPHOS)/CI_pyr_-OXPHOS; (ii) proline contribution = (CI + ProDH-OXPHOS − CIc-OXPHOS)/CIc-OXPHOS; (iii) succinate contribution = (CI + ProDH + CII-OXPHOS − CI + ProDH-OXPHOS)/CI + ProDH-OXPHOS; (iv) and G3P contribution = (CI + ProDH + CII + mtG3PDH-OXPHOS − CI + ProDH + CII-OXPHOS)/CI + ProDH + CII-OXPHOS). Injection of carbonyl cyanide 4-(trifluoromethoxy)phenylhydrazone (FCCP, steps of 0.5–1 μM), was then performed to measure the noncoupled respiration i.e., the nonphosphorylating respiration stimulated to maximal oxygen consumption (CI + ProDH + CII + mtG3PDH-ETS), and calculate the E_max_/P_max_ ratio (CI + ProDH + CII + mtG3PDH-ETS/ CI + ProDH + CII + mtG3PDH-OXPHOS) which indicates a possible limitation of the phosphorylation system if higher than 1.0 [19,45]. Subsequent inhibitions of complexes I, II, and III by rotenone (0.5 μM), malonate (5 mM), and antimycin A (2.5 μM) were performed to evaluate the residual oxygen consumption, which was used to correct the previous respiration rates measured. All measurements are presented as means of mass-specific respiration rates expressed as pmol O_2_·s^−1^·mg^−1^ of permeabilized fibers ± s.e.m.

### 4.5. Enzymatic Activities

Enzymatic activities were determined using a BioTek Synergy H1 microplate reader set at 24 °C (BioTek^®^, Montréal, QC, Canada). For each genotype, 6 pools of 5 flies were used (N = 6). The flies were homogenized in phosphate-buffered saline using a pellet pestle, and the resulting homogenates were centrifuged at 750× *g* for 5 min at 4 °C. The supernatant was then directly used for measurements of PK, LDH, ALAT, PDH, CS, and HOAD, following protocols already established [46], except for ALAT activity. The protocol for ALAT activity was modified from [47] by following the disappearance of NADH at 340 nm for 4 min in a reaction medium containing 100 mM potassium phosphate, 0.32 mM NADH, 10 mM α-ketoglutarate, 500 mM alanine, 0.10 mM pyridoxal phosphate, and 0.6 U·mL^−1^ lactate dehydrogenase, pH 7.2. Enzymatic activities were normalized to protein concentration, which was determined using the bicinchoninic acid method [48], with bovine serum albumin as standard, and expressed as mU·mg^−1^ protein, where U represents 1 μmol of substrate transformed to product in 1 min.

### 4.6. Relative Metabolites Abundance

Hydrophilic metabolites were extracted by homogenizing 21 flies in a 1:1 acetonitrile:distilled water solution. The homogenates were then centrifuged at 13,500 rpm for 10 min at 4 °C to remove proteins and debris. The supernatant was collected, transferred to a glass test tube, and frozen at −80 °C for at least 24 h. The samples were then thawed at room temperature and evaporated under nitrogen in a lightly heated water bath. The internal standard 4,4-dimethyl-4-silapentane-1-sulfonic acid (DSS) was then added to the sample (0.5 mM of DSS final concentration) and the resulting mix was solubilized in 700 µL of deuterium oxide. The ^1^H-NMR spectra for the extracted metabolites, dissolved in H_2_O:D_2_O (9:1), were recorded on a Bruker Advance III 400 MHz spectrometer at 298 °K equipped with a 5mm TCI CryoProbe. The noesypr1d pulse sequence was used. A total of 128 scans of 64,000 data points were recorded with a recycle delay of 1 s per scan. The spectral width was set at 12 KHz and the acquisition time at 6.6 s. The obtained spectra (N =6–7 for each genotype) were analyzed using the Chenomx NMR Suite (Chenomx Inc., Edmonton, AB, Canada) and the Human Metabolome DataBase (HMDB, Edmonton, AB, Canada). A series of 49 hydrophilic metabolites were identified and included in the analysis (Table S2).

### 4.7. Climbing Assay

Climbing ability (negative geotaxis) was measured as previously described [15]. Briefly, flies were transferred to empty vials (9.4 cm tall × 2.5 cm wide), divided in four quadrants, and were tapped to the bottom of the vial (N ≥ 75 for each genotype). The number of flies that climbed into the different quadrants (0–25, 25–50, 50–75, and 75–100% of the total height of the vial) was noted after 30 s of climbing. These assays were repeated three times for each vial.

### 4.8. Statistical Analysis

Statistical analyses were performed with R software (version 3.1.0, Free Software Foundation, Boston, MA, USA) and MetaboAnalyst 4.0 [20]. For mitochondrial oxygen consumption, enzymatic activities, relative metabolite abundance, and climbing assay, Student’s t-tests were performed to determine specific differences between the genotypes after normality (Shapiro-Wilk) was verified and a comparison of variances (F-test) was performed. For comparisons of EC_50_ for mitochondrial pyruvate carrier inhibition, an estimated ratio of effect doses, followed by calculating differences between parameter estimates, were performed using the EDcomp and compParm functions of the ‘drc’ package [49]. The relative metabolite abundance was further analyzed using a partial least squares discriminant analysis (PLS-DA) in MetaboAnalyst 4.0 after sample normalization with DSS, and after data log transformation and auto scaling (i.e., mean-centered and divided by the standard deviation of each variable) in order to minimize possible differences in concentration between samples. The PLS-DA identified the metabolites driving the separation and/or clustering among genotypes by ascribing a variable importance to the projection score (VIP).

## Supplementary Materials

The following are available online at https://www.mdpi.com/2218-1989/10/9/363/s1, Table S1: Primer sequences used for q RT-PCR, Table S2: Relative metabolites abundance and ascribed VIP scores assigned with PLS-DA identifying the metabolites driving the separation and/or clustering among genotypes.
