# Supplementary material for: Metabolic Characterization and Consequences of Mitochondrial Pyruvate Carrier Deficiency in Drosophila melanogaster
Source: Metabolites. 2020 Sep 6;10(9):363. doi: 10.3390/metabo10090363 (PMC7570025; doi:10.3390/metabo10090363)
Supplement: Supplementary file 1 [file metabolites-10-00363-s001.pdf]

Metabolic characterization and consequences of mitochondrial pyruvate carrier deficiency in *Drosophila melanogaster*.

Chloé Simard[1], Andréa Lebel[1], Eric Allain[2], Mohamed Touaibia[1], Etienne Hébert-Chatelain[3] and Nicolas Pichaud[1]*

[1] Department of Chemistry and Biochemistry, Université de Moncton, Moncton, NB, Canada, E1 A 3E9.

[2]Atlantic Cancer Research Institute (ACRI), Moncton, NB, Canada, E1C 8X3.

[3]Department of Biology, Université de Moncton, Moncton, NB, Canada, E1 A 3E9.

* Correspondence: nicolas.pichaud@umoncton.ca

Supplementary material

Table S1. Primer sequences used for q RT-PCR.

Gene		Primer Sequence (5'-3')	Accession nº
MPC1	F	ctcaaaggagtggcgggatt	NM_001275791
	R	cagggtcagagccaatgtca	
α-tubulin	F	cgtttgtcaagcctcatagc	NM_057424.4
	R	atggatagagatacattcacgcata	
RpL32	F	tggtttccggcaagcttca	NM_170461.3
	R	tgttgtcgatacccttgggc	

Table S2. Relative metabolites abundance and ascribed VIP scores assigned with PLS-DA identifying the metabolites driving the separation and/or clustering among genotypes.

**Metabolites**	**Relative abundance (mM)**		**VIP score**
	**WT**	**MPC1^{def}**	
β-Alanine	0.2508 ± 0.0155	0.2645 ± 0.0326	0.057875
3-Hydroxybutyrate	0.3153 ± 0.0685	0.1443 ± 0.0255	1.5111
3-Phosphoglycerate	1.1571 ± 0.2041	0.6963 ± 0.1628	1.0191
Acetate	0.0702 ± 0.0131	0.0712 ± 0.0052	0.31614
Acetoacetate	0.0251 ± 0.0050	0.0158 ± 0.0017	1.2023
Alanine	0.1882 ± 0.0273	0.1368 ± 0.0283	0.82697
Argininosuccinate	0.3835 ± 0.0651	0.1868 ± 0.0618	1.3493
Asparagine	0.0586 ± 0.0133	0.0238 ± 0.0037	1.482
Carnitine	0.0276 ± 0.0051	0.0247 ± 0.0062	0.21399
Citrate	0.0139 ± 0.0033	0.0064 ± 0.0014	1.1585
Citrulline	0.1185 ± 0.0175	0.0498 ± 0.0090	1.6642
Aspartate	0.0340 ± 0.0077	0.0173 ± 0.0053	1.1808
Fructose	1.8084 ± 0.2155	1.2147 ± 0.2837	1.1209
Glutamine	0.5443 ± 0.0312	0.3761 ± 0.0843	1.181
Ethanol	0.0599 ± 0.0142	0.0183 ± 0.0043	1.5738
Fructose 6-phosphate	0.1210 ± 0.0178	0.0503 ± 0.0135	1.4129
Fumarate	0.0051 ± 0.0004	0.0048 ± 0.0016	0.72343
Glucosamine 6-phosphate	0.0943 ± 0.0079	0.1192 ± 0.0184	0.74216
Glucose	0.3911 ± 0.0617	0.3374 ± 0.0550	0.41908
Glucose-6-phosphate	0.1318 ± 0.0271	0.1232 ± 0.0104	0.2664
Glutamate	0.1451 ± 0.0153	0.0992 ± 0.0150	1.2049
Glycerol	0.1443 ± 0.0334	0.1149 ± 0.0276	0.2679
Glycerol 3-phosphate	0.4279 ± 0.0489	0.2784 ± 0.0543	1.2602
Glycine	0.0231 ± 0.0056	0.0264 ± 0.0055	0.31072
Glycogen	13.7128 ± 2.9518	8.8687 ± 2.0442	0.5255
Isocitrate	0.0458 ± 0.0060	0.0356 ± 0.0047	0.87803
Isoleucine	0.0179 ± 0.0034	0.0113 ± 0.0019	1.0122
Lactate	0.0513 ± 0.0080	0.0296 ± 0.0089	1.2699
Arginine	0.0325 ± 0.0069	0.0238 ± 0.0027	0.58548
Cysteine	0.1300 ± 0.0301	0.0732 ± 0.0157	0.94576
Leucine	0.0233 ± 0.0028	0.0176 ± 0.0032	0.89588
Proline	0.0754 ± 0.0093	0.0535 ± 0.0079	1.0469
Lysine	0.0528 ± 0.0122	0.0304 ± 0.0052	0.81582
Malate	0.0793 ± 0.0224	0.0359 ± 0.0078	1.0141
Ornithine	0.0396 ± 0.0070	0.0204 ± 0.0033	1.3317
Oxaloacetate	1.7622 ± 0.2368	1.3291 ± 0.1468	0.86807

Phenylalanine	0.0164 ± 0.0043	0.0112 ± 0.0032	0.80732
Phosphoenolpyruvate	0.0786 ± 0.0147	0.0625 ± 0.0197	0.65359
Propionate	0.0316 ± 0.0047	0.0236 ± 0.0030	0.77345
Pyruvate	0.0134 ± 0.0015	0.0085 ± 0.0012	1.3816
Serine	0.0952 ± 0.0197	0.0961 ± 0.0199	0.37377
Succinate	0.0750 ± 0.0051	0.0638 ± 0.0102	0.84503
Sucrose	0.0246 ± 0.0042	0.0170 ± 0.0029	0.96644
Taurine	0.1966 ± 0.0140	0.1451 ± 0.0213	1.2444
Threonine	0.0742 ± 0.0130	0.0249 ± 0.0062	1.6898
Trehalose	0.0181 ± 0.0057	0.0181 ± 0.0052	0.059345
Tyrosine	0.0208 ± 0.0055	0.0161 ± 0.0041	0.35208
UDP-glucose	0.0259 ± 0.0027	0.0309 ± 0.0032	0.75491
Valine	0.0118 ± 0.0027	0.0072 ± 0.0013	0.84082
